# Association between Retinal Arteriolar and Venule Calibre with Prevalent Heart Failure: A Cross-Sectional Study

**DOI:** 10.1371/journal.pone.0144850

**Published:** 2015-12-11

**Authors:** Kevin Phan, Paul Mitchell, Gerald Liew, Adam J. Plant, Sarah B. Wang, Cheryl Au, Joseph Chiha, Pramesh Kovoor, Aravinda Thiagalingam, George Burlutsky, Bamini Gopinath

**Affiliations:** 1 Centre for Vision Research, Department of Ophthalmology and Westmead Millennium Institute, University of Sydney, Sydney, New South Wales, Australia; 2 Centre for Heart Research, Westmead Millennium Institute, University of Sydney, NSW, Australia; University of Florida, UNITED STATES

## Abstract

**Background:**

There is evidence to suggest that microvascular disease, particularly diabetic retinopathy, plays a role in the pathogenesis of HF. However, whether changes in retinal vessel calibre predicts HF is unclear. The purpose of this study was to examine the association of retinal microvascular structure with prevalent heart failure (HF).

**Methods:**

The Australian Heart Eye Study (AHES) is a cross-sectional study that surveyed 1680 participants who presented to a tertiary referral hospital for the evaluation of potential coronary artery disease by coronary angiography. Retinal vessel calibre was graded using retinal photography and participants’ self-reported echocardiography-confirmed HF was obtained via an extensive medical questionnaire.

**Results:**

There were 107 participants (8.1%) with prevalent self-reported HF. Persons with wider retinal arteriolar calibre (comparing highest versus lowest tertile or reference) were more likely to have prevalent HF (OR 3.5; 95% CI, 1.7–7.2) when adjusted for age and sex. After further adjustment for body mass index, hypertension, diabetes, smoking status, triglycerides and estimated glomerular filtration rate, this association remained significant (OR 4.5; 95% CI, 2.0–9.8). After further stratification, this association remained significant among participants with diabetes (OR 10.3; 95% CI, 2.7–39.3) but not in those without diabetes (OR 2.7; 95% CI, 0.9–7.5). The strength of this association was not dependent on the length of history of diabetes, or retinopathy status. There was no significant association between retinal venular calibre and prevalence of HF.

**Conclusions:**

Wider retinal arteriolar diameter was significantly and independently associated with prevalent HF in participants of a cross-sectional study. This association was significant stronger among participants with diabetes compared to without diabetes. No association was found between retinal venule calibre with prevalent HF.

## Introduction

Chronic heart failure (HF) is one of the major causes of mortality and morbidity in the Western world, occurring in more than 5 million individuals and responsible for more than 1 million hospital studies.[1] While the exact mechanisms underlying the pathophysiology of HF remains unclear, it has been suggested that HF is associated with coronary artery disease (CAD)[2] and hypertension.[3] However, some individuals without CAD or hypertension may still be predisposed to HF with poor prognosis and outcomes, indicating that other pathological processes could be involved. In particular, diabetes has been associated with increased incidence of heart failure.[4] Microvascular disease has been implicated in diabetic cardiomyopathy and HF,[5, 6] even in patients without angiographically detectable CAD or present asymptomatically.

Several studies have supported the integral role of microvascular disease in the pathogenesis of HF in the general population. The Atherosclerosis Risk In Communities (ARIC) study demonstrated an independent association between retinopathy and congestive HF,[7] even after exclusion of patients with CAD, diabetes or hypertension. This association remained significant at the 9-year follow-up, and after further adjustment for known cardiovascular risk factors, glycemic control, carotid atherosclerosis and serum markers of endothelial function.[8] While retinal arteriolar dilation has been shown to predict incident retinopathy in diabetic individuals, there is limited evidence investigating the relationship between changes to the retinal vessel calibre and HF.

Potential links between retinal vessel calibre and HF would have important clinical implications, as individuals with early changes in the retinal microvasculature could be screened or monitored for development of HF. Retinal vessel calibre changes can be measured directly and appeals as a non-invasive test and potential marker for underlying cardiac disease. We therefore aimed to assess the independent association between retinal arteriolar and venule calibre with prevalent HF, in a cross-sectional study–Australian Heart Eye Study (AHES).

## Methods

Ethics approval was obtained from Western Sydney Local Health Network Human Research Ethics Committee. Written informed consent was obtained from all participating patients via a patient information sheet explaining the AHES objectives, the role of the research participants, risks, costs, potential benefits and other relevant information, as well as the signed consent form. The Western Sydney Local Health Network Human Research Ethics Committee approved the informed consent procedure.

### Participants

The AHES is a cross-sectional study of 1680 participants who presented to Westmead Hospital (Sydney, Australia), between June 2009 and January 2012 to evaluate potential CAD by coronary angiography. All eligible patients presenting for assessment of suspected CAD were included in this study. All patients approached for the present study gave consent for invasive coronary angiography. Relative contraindications for invasive coronary angiography included pre-existing renal impairment and contrast allergy. However, these were not absolute and coronary angiography was performed if the risk benefit ratio was favourable. Most patients were recruited following their angiogram. As these patients are hospitalised, and investigations are performed prior to angiography on a routine basis, information collected by medical personnel was also included in the study.

Examinations and measurements included a detailed medical history questionnaire, visual acuity testing, biochemical, angiographic, peripheral arteriolar wave form analysis, pulse wave analysis, ankle-brachial pressure index, peripheral blood pressure measurements, invasively measured blood pressure measurements, transthoracic echocardiography, electrocardiography, blood count analysis, genetic testing and retinal photography data. If there was incomplete information of retinal vessel calibre, and patient status of diabetic retinopathy or age-related macular degeneration (AMD), these patients were excluded. Ethics approval was obtained from Western Sydney Local Health Network Human Research Ethics Committee.

### Medical History

A 252-item questionnaire was designed and used to obtain patient medical history, cardiovascular and familial risk factors. The questionnaire forms were collected and entered by two study personnel with single entry. The forms included current cardiac rhythm status, previous/recent history of angina, previous myocardial infarction, previous angiography, previous intervention (coronary artery bypass grafting, or percutaneous coronary artery stenting), previous stroke, transient ischemic attack (TIA), hypertension, hypercholesterolemia, diabetes mellitus, and current management of these chronic conditions, current medications, smoking status and alcohol consumption.

### Prevalence of heart failure

A comprehensive questionnaire was used by study personnel to obtain a detailed medical history, including year of heart failure diagnosis, classification of heart failure (NYHA Class I-IV), and treatment for heart failure including fluid restriction (liters per day), biventricular pacemakers, and medications. HF prevalence was defined as a positive response to both questions "When was it diagnosed?" and “Was diagnosis confirmed with transthoracic echocardiography or transoesophageal echocardiography”. Other information collected included past cardiovascular events, cardiovascular risk factors, other medical conditions, drug and alcohol history, and history of past angiography and/or interventions (coronary artery stent or coronary artery bypass graft).

### Retinal vessel calibre assessment

Retinal photography was used to assess retinal vessel calibre. Participants had dilated, digital photographs taken of the optic disc and macula of both eyes using a pre-calibrated Canon 60° fundus camera (Model CF-60DSi, Canon Inc., Tokyo, Japan) with an attached digital camera (Model 1DSmklll, Canon Inc., Tokyo, Japan). Retinal vessel calibre measurements for the right eye of each participant were used. Left eye measurements were used if right-eye photographs were un-gradable. One grader, masked to participant identity and characteristics, measured retinal vessel caliber using a computer-assisted program (IVAN, University of Wisconsin, Madison) with high reproducibility, this has been previously described[9, 10]. The width (diameter) of all retinal arterioles and venules coursing through a pre-specified region of the retina are measured. These values are combined to obtain average retinal arteriolar or venular width for that eye using the Parr-Hubbard formula as modified by Knudtson et al,[11] and presented as central retinal arteriolar equivalent (CRAE) or central venular equivalent (CRVE).

### Assessment of covariates

Diastolic and systolic blood pressures and heart rate were measured, with a single reading, from the right arm with an Intellisense^TM^ OMRON digital automatic blood pressure monitor in the supine position (Model HEM-907; OMRON Healthcare, Singapore). Invasive blood pressure measurements were performed using a fluid filled catheter in the central aorta and measured with a Mac-Lab hemodynamic system (GE Healthcare Milwaukee, WI). Biochemical data, including renal function (creatinine), eGFR, full blood counts, cardiac enzyme (including creatinine kinase, 4th and 5th generation Troponin T levels), fasting blood sugar, HbA1c level, fasting cholesterol levels, and thyroid function tests were collected from the participant’s medical record. Diabetes status was determined as per self-reported diabetes history and current use of diabetic medications. Patients with unknown diabetes status were excluded from analysis.

### Statistical analysis

All analyses were performed using SAS statistical software (version 9.2, SAS Institute Inc., Cary, North Carolina, USA). Statistical significance was defined as P-value < 0.05.

Multivariable analyses using logistic regression models adjusted for cardiovascular risk factors including age, sex, BMI, hypertension, diabetes mellitus, smoker, eGFR, were used to estimate odds ratios (OR) and 95% confidence intervals (CI), in order to determine whether associations exist between retinal vessel caliber (analyzed as tertiles) and prevalent HF. Adjustment factors were choosen based on confounders which significantly modified the effect of retinal vessel caliber in age-sex adjusted models in relation to prevalent HF, or significantly predicted the outcome variable of prevalent HF. In analysis of the potential association between retinal vessel caliber and prevalent HF stratified by diabetes status, the same cardiovascular risk factor covariates (except for diabetes) was adjusted for in multivariable analyses.

If a significant association was found between retinal microvasculature and prevalent HF, subgroup analysis was performed to see if the association held in (1) diabetic versus non-diabetic patients, and (2) when patients with cardiovascular disease (CVD, defined by history of myocardial infarction, angina or stroke) and CKD (eGFR < 60) were excluded from analysis.

## Results

Participants with prevalent HF were more likely to be older, hypertensive, have higher BMI, diabetes and lower eGFR (Table 1). Patients without HF had a higher prevalence of smoking. 365 participants were excluded from the present analysis due to missing data. From a cross-sectional analysis of the remaining 1315 patients, 107 (8.1%) participants were identified to have prevalent HF.

**Table 1:** Baseline characteristics of patients with and without self-reported echocardiography confirmed-heart failure.

	No HF (n = 1208)	HF (n = 107)	P-value
Age	61.27±11.54	65.36±10.44	0.0004
Male (%)	912 (75.50)	75 (70.09)	0.22
BMI	29.39±5.61	31.13±6.96	0.02
Systolic blood pressure (mmHg)	128.25±19.44	127.93±22.90	0.89
Diastolic blood pressure (mmHg)	73.40±12.16	71.21±14.01	0.12
Hypertension (%)	840 (69.94)	96 (89.72)	<0.0001
Diabetes mellitus (%)	475 (39.40)	54 (50.47)	0.03
Smoker (%)	320 (26.56)	18 (16.98)	0.033
HDL (mmol/L)	1.05±0.30	1.00±0.32	0.36
Triglycerides (mmol/L)	1.77±0.97	1.58±0.92	0.16
eGFR (mL/min/1.73m^2^)	86.64±25.32	73.57±26.10	<0.0001

HF, heart failure; BMI, body mass index; HDL, high density lipoprotein; eGFR, estimated glomerular filtration rate

After initial adjustments for age and sex (Table 2), participants in the highest versus lowest (reference) tertile of retinal arteriolar calibre had 3.55-fold higher odds of having heart failure. After further adjustment for cardiovascular risk factors, participants with the widest compared to narrowest retinal arteriolar calibre had a 4.46-fold higher odds of self-reporting prevalent HF. When retinal arteriolar calibre was analyzed as a continuous variable, the association remained significant (OR, 2.18; 95% CI, 1.46–3.28; P = 0.0002). There was no significant association between retinal venular calibre and prevalence of HF analysing venular calibre in tertiles (Table 2) or as a continuous variable (OR 0.996; 95% 0.66–1.49; P = 0.98).

**Table 2:** Relationship between retinal arteriolar calibre and prevalent heart failure.

Subgroup	Number	No (%) affected by HF	Age-sex adjusted OR (95% CI)	Multivariate* adjusted OR (95% CI)
Retinal arteriolar calibre (μm)				
1^st^ tertile (≤141.87)	286	14 (4.90)	1.00 (reference)	1.00 (reference)
2^nd^ tertile (141.90–153.71)	262	13 (4.96)	1.26 (0.57–2.78)	1.40 (0.59–3.34)
3^rd^ tertile (≥153.75)	280	30 (10.71)	3.55 (1.75–7.21)	4.46 (2.03–9.78)
P-value for trend			P = 0.0003	P = 0.0001
Retinal venular caliber (μm)				
1^st^ tertile (≤213.84)	272	24 (8.82)	1.00 (reference)	1.00 (reference)
2^nd^ tertile (214.02–232.87)	280	15 (5.36)	0.71 (0.36–1.41)	0.89 (0.43–1.84)
3^rd^ tertile (≥232.91)	273	18 (6.59)	1.04 (0.53–2.07)	1.01 (0.45–2.28)
P-value for trend			P = 0.9660	P = 0.9820

*Multivariate adjusted models included the covariates age, sex, body mass index (BMI), hypertension, diabetes mellitus, eGFR and smoking status; HF, heart failure; OR, odds ratio; CI, confidence interval

Stratified analyses showed that the association between wider retinal arteriolar diameter (highest versus lowest tertile) and a greater likelihood of prevalent HF was more marked among those with diabetes (OR, 10.32; 95% CI, 2.714–39.26) than in those without diabetes (p = 0.0006) (Table 3). The strength of this association in diabetic participants was not significantly different when stratified into length of diabetes history (≤5, 6–10, 11–20, ≥20 years; P = 0.2744). Furthermore, the association was not different between diabetic patients without retinopathy versus diabetics patients with retinopathy (P = 0.3922). The association between retinal arteriolar calibre and prevalent heart failure was also stratified according to hypertension status. The association remained significant when considering only hypertensive patients only (P-trend for tertiles, P = 0.0009).

**Table 3:** Association between retinal arteriolar caliber and prevalent heart failure according to diabetes status.

	No Diabetes		Diabetes	
Subgroup	Multivariate* adjusted OR (95% CI)	P-value	Multivariate* adjusted OR (95% CI)	P-value
Retinal arteriolar calibre (μm)				
1^st^ tertile (≤141.87)	1.0 (reference)	NA	1.0 (reference)	NA
2^nd^ tertile (141.90–153.71)	0.72 (0.21–2.52)	0.61	3.05 (0.80–11.64)	0.10
3^rd^ tertile (≥153.75)	2.68 (0.97–7.47)	0.16	10.32 (2.71–39.26)	0.0006
P-value for trend	P = 0.2137		P = 0.0659	

*Multivariate adjusted models included the covariates age, sex, body mass index (BMI), hypertension, eGFR and smoking status; OR, odds ratio; CI, confidence interval; NA, not available

## Discussion

In the present cross-sectional study AHES, we report an association between wider arteriolar retinal diameter and prevalent heart failure among participants with diabetes. Patients with wider arteriolar retinal calibre in the highest tertile were 4.5 times more likely to have HF compared to participants with narrower retinal arteriolar diameters in the lowest tertile, even after controlling for conventional cardiovascular risk factors. The association was stronger in those with diabetes, who had over 10-fold higher odds of having HF. No significant association was found between retinal venular calibre and prevalent HF in the AHES cohort.

Retinal microvascular signs have inconsistently been shown in the literature to be associated with systemic cardiovascular disease [12–16]. Changes in retinal microvasculature may reflect subclinical pathological changes prior to the development of cardiovascular disease and diagnosis. In the Multi-Ethnic Study of Atherosclerosis (MESA), a cross-sectional study of 5979 participants demonstrated significant associations between retinal arteriolar and venular calibre with hypertension, diabetes, obesity measures, and dyslipidemia.[17] Narrower retinal arterioles and wider venules have also been reported to be associated with increased risk of CAD in females,[18] and increased risk of mortality due to cardiovascular causes.[19] There is also evidence demonstrating 3-fold increased risk of stroke in healthy individuals with narrow retinal arteriolar calibre and retinopathy. The evidence for the integral role retinal microvascular changes in the pathophysiology of cardiovascular disease implicates the potential role for simple, non-invasive monitoring of the human retinal microcirculation for early intervention.

Few studies have explored the role of retinal microvascular disease in other cardiovascular diseases such as congestive HR. One of the few studies to explore this relationship is the Atherosclerosis Risk in Communities (ARIC) study, a prospective study of four US communities. The 7-year cumulative incidence of HF was 5.4%, and retinopathy was found to be independently associated with a 3-fold increased risk of HF.[7, 8] While other retinal vessel calibre changes have been shown to significantly predict retinopathy, no study to the best of our knowledge has investigated directly the relationship between retinal microvascular structure and HF. The present findings demonstrate for the first time that retinal arteriolar widening is significantly associated with prevalent HF, and this association remained significant only among participants with diabetes.

The mechanisms underlying the relationship between retinal microvascular structural changes and prevalent HF are not well established. However, the biological rationale for an association between retinal arteriolar widening and HF is plausible. It is reasonable to expect that there is a continuum of microvascular structural adaptations which represent overall microvascular damage or inflammation in an individual, progressing form early retinal arteriolar widening, to retinopathy and finally HF. Even as early as 1939, it was recognised that the relationship between retinal vessel changes and cardiovascular risk and mortality was dose-dependent.[20] The “continuum” hypothesis is supported by results from the ARIC population study, which showed that both mild and severe retinopathy conferred higher risk for HF,[8] with stronger associations observed in the severe retinopathy group. Retinal arteriolar widening has also been shown to be associated with retinopathy by several population studies.[21, 22] A similar relationship was also investigated in the Multi-ethnic Study of Atherosclerosis (MESA), a prospective multi-ethnic population cohort who initially did not have baseline cardiovascular disease[23]. The MESA study demonstrated a significant association between wider retinal arteriolar calibre and increased risk of developing diabetes. Interestingly, the tertile cut-offs for retinal arteriolar calibre in this study was very similar to that reported in the present study, suggesting that the absolute size of the retinal vessels in the present study population who may potentially have coronary artery disease is comparable with that of a general non-clinical population. Furthermore, similar associations have also been found in several other cross-sectional data reported in different population studies[24–26]. While the underlying mechanisms are not clear, retinal arteriolar widening may be indicative of a combination of tissue hypoxia and impaired vasogenic autoregulation, which may overall be a surrogate marker of early onset systemic microvascular disease such as diabetes and retinopathy[22, 27]. As such, early changes in retinal arteriolar calibre may be a predictor of long-term increase burden on the heart and manifest as compromised cardiac function in terms of ventricular emptying and cardiac output.

To test the continuum hypothesis, we stratified our analysis according to length of diabetes history and retinopathy status, with stronger associations expected in participants with longer history of diabetes and with diabetic retinopathy. No significant association was demonstrated, which may be due to the small number of patients with prevalent heart failure and retinopathy in our cohort. Consequently, the statistical power may have been inadequate to detect any actual differences between these subgroups.

Current guidelines indicate routine screening for retinopathy in patients with diabetes. Multiple studies have also emphasised the importance of using retinal visualization for risk stratification in coronary artery disease, to enable more effective primary prevention strategies. However, few international guidelines have emphasised the role of screening for early retinal vessel calibre changes in this particular population subset. Our results suggest that patients with diabetes with evidence of significant narrowing of retinal arteriolar calibre could also benefit from cardiovascular examinations during follow-up. This could potentially help identify subclinical HF[28, 29], thus allowing for earlier management and intervention.

Strengths of the present study include the use of a relatively large population-based sample with high participation rates, as well as objective measurements of visual acuity and retinal calibre assessment using fundus photography. Retinal grading systems used were standardized and comparable to the grading systems employed in other prominent population-based studies in this field including the Beaver Dam Eye Study and the AIC. Other strengths including adjustment for major confounders and traditional cardiovascular risk factors, as well as stratification into diabetes versus no diabetes subgroups.

The present study results are also constrained by several limitations. First, the echocardiography confirmed HF was obtained by self-report from participants, and the possibility of selection bias or underestimation of prevalent HF cannot be discounted. The AHES population is based on participants who underwent angiography for potential coronary artery disease at a tertiary hospital center. As such, the population was not randomly selected, and consisted of a higher proportion of hyperlipidaemic, diabetic and hypertensive male population. As such, caution should be taken when interpreting the present results, especially when considering other population subsets such as younger, healthy females. Hypertensive status wa also defined based on medical questionnaire responses or a single blood pressure test, which has limited validity and may undermine confidence in the results. A small number of patients were found to have HF, which resulted in larger confidence intervals when the relationship was stratified according to diabetes status. Furthermore, given that all patients were selected for coronary angiography, patients with positive coronary disease would theoretically have smaller arteriolar calibre. The cut-off values for retinal vessel calibre tertiles in this study were slightly lower compared to prior studies in non-angiography populations[19], thus potentially contributing risk of bias to the presented results.

Given the cross-sectional design of the AHES, no incident HF data was available for analysis. Thus, a definitive judgement regarding the risk of developing HF and its relationship with retinal arteriolar calibre cannot be concluded. Another unaccounted limitation is that HF patients may be on high doses of vasodilator medications, which may have affected the presented trends. Future prospective population-based studies should be designed to assess whether this association holds after long-term follow-up. Current trends observed in this study should be confirmed by future larger prospective, adequately powered population studies. Although multivariable adjusted models were used to account for traditional cardiovascular risk factors, the effects of the differential use of anti-hypertensive agents and hypoglycaemic agents may confound the presented results.

## Conclusion

In summary, we report a significant association between wider retinal arteriolar vessels and prevalent HF in individuals who presented to angiography for testing for potential coronary artery disease, independent of traditional cardiovascular risk factors. This association was stronger in participants with diabetes compared to without diabetes. These findings add further support to the potential role of the retinal microvasculature and disease in aetiology of HF in diabetes. Patients with diabetes with adverse retinal microvascular signs, particularly retinal arteriolar calibre dilation, may warrant more careful cardiovascular evaluation and follow-up.
